# Refining the genome of alkylbenzene-degrading *Rhodococcus* sp. DK17 and comparative analysis with genomes of its deletion mutants

**DOI:** 10.1128/mra.01134-24

**Published:** 2025-02-25

**Authors:** Jehyun Jeon, Dockyu Kim, Eungbin Kim, Yung Mi Lee

**Affiliations:** 1 Division of Life Sciences, Korea Polar Research Institute, Incheon, South Korea; 2 Department of Systems Biology, Yonsei University, Seoul, South Korea; Rochester Institute of Technology, Rochester, New York, USA

**Keywords:** alkylbenzene degradation, *Rhodococcus*, UV-induced mutant, genome

## Abstract

*Rhodococcus* sp. strain DK17 degrades various alkylbenzenes, including *o*-xylene, making it a potential biocatalyst for the production of fine chemicals. DK17 carries the degradative genes on linear mega-plasmids. Here, we present the refined DK17 genome and analyze the genetic variations in UV-induced mutants DK176 and DK180.

## ANNOUNCEMENT

*Rhodococcus* sp. DK17 was isolated from surface soil collected using a steel spatula and pooled into a plastic tube at a crude oil-contaminated site (Yeocheon Industrial Complex, Korea). DK17 can utilize *o*-xylene and various monocyclic aromatics as sole carbon and energy sources in minimal salt basal (MSB) medium (1, 2). DK17 carries three linear mega-plasmids (pDK1–3), with the *o*-xylene degradation gene cluster, *akb*, on pDK2 (3, 4). UV-induced mutants DK176 and DK180 lost the ability to degrade *o*-xylene due to the loss of pDK2 and a nonsense mutation in a benzene ring cleavage enzyme gene, respectively (1). A previous draft genome of DK17 (BioProject PRJNA157361) revealed 9.1 Mb in 135 contigs within 27 scaffolds (5). However, incomplete genome for DK17 and the lack of genomic information of DK176 and DK180 hindered understanding the structure of mega-plasmids and alkylbenzene degradation. To clarify the genetic basis behind alkylbenzene degradation loss, genome resequencing and comparison of DK17, DK176, and DK180 were performed.

Strains were grown on MSB agar with 5 mM glucose at 30°C for 3 days, and genomic DNA was extracted using a G-spin Genomic DNA Extraction Kit (iNtRON Biotechnology, Korea). Libraries were prepared using the SQK-LSK110 protocol (Oxford Nanopore Technologies) and TruSeq DNA PCR-free protocol (Illumina) from 1 µg of unsheared DNA per sample, with size selection performed using AMPure XP beads.

Sequencing was performed on Oxford Nanopore GridION using an FLO-MIN106 R9.4.1 flow cell and on an Illumina NovaSeq (151 bp paired-end reads). Base calling for Nanopore long reads was performed using MinKNOW (v22.08.9) and Guppy (v6.2.11) with the high accuracy model. For pre-processing, NanoFilt (v2.6.0) (6) with -q 10 -l 5000 and Trimmomatic (v0.21.0) (7) were used for Nanopore and Illumina reads, respectively. Hybrid assembly and circularization of the replicons, distinguishing between linear and circular forms, were performed with Unicycler (v0.4.8-beta) (8). Genome completeness was evaluated using BUSCO (v5.0.0) (9) with bacteria_odb10 library, and annotation was performed using Prokka (v1.13.5) (10). Genome synteny was analyzed using BLASTn (11) with options of -evalue 1e-05. Default parameters were applied unless specified.

**Fig 1:**
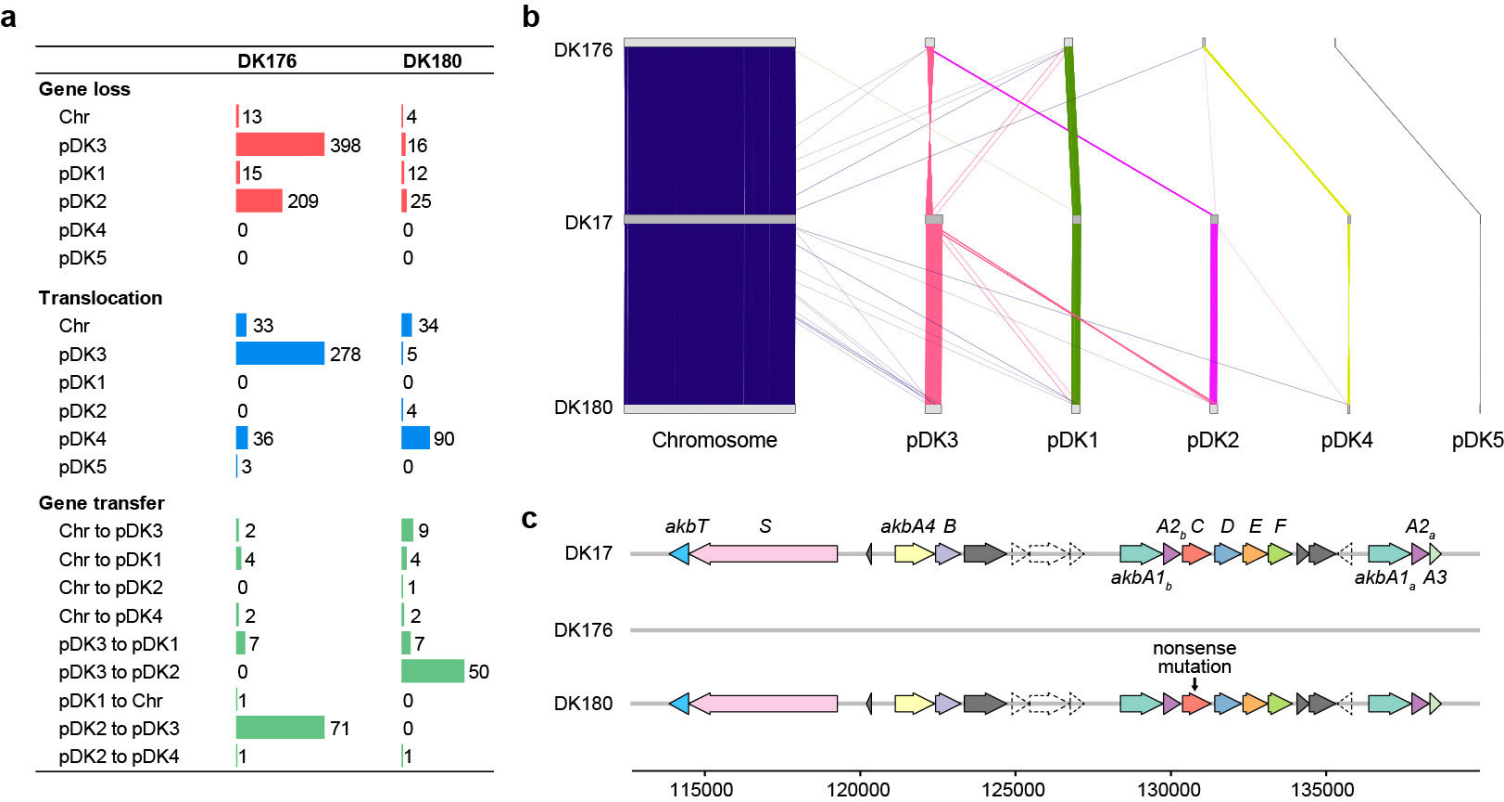
Comparisons of genetic variations in three *Rhodococcus* genomes: DK17 (wild type), DK176 (UV-induced mutant strain), and DK180 (UV-induced mutant strain). (a) The number of genetic variations in DK176 and DK180 compared with DK17. (b) Lines connecting chromosomes and plasmids indicate syntenic genes, colored by association with those of DK17. The plot was visualized using the syntenyPlotteR package (v1.0.0) in R (v4.3.3). (c) Gene organization on pDK2 in three genomes. Putative genes are colored by dark gray and pseudogenes are represented by a dashed arrow shape.

The detailed statistics and genetic variations of genomes are summarized in Table 1 and Fig. 1a. In DK17, each of pDK1, pDK2, and pDK3 possesses genes for telomere-binding and terminal proteins, essential for linear plasmid maintenance (12). Differences in genome sizes (8.8 to 9.5 Mb) resulted from the loss of genes (635 in DK176 and 57 in DK180) compared with DK17 (Fig. 1b). Notably, DK176 completely lost *akb* cluster with pDK2 (Fig. 1c) along with one-half deletion of pDK3, while DK180 showed partial deletions of pDK2 and pDK3. Frequent genetic changes, including translocation, gene transfer, and gene loss, particularly on pDK2 and pDK3, were observed in the mutants. These findings suggest that DK17 may adapt, evolve, or survive under unfavorable environmental conditions, such as UV exposure and high temperatures, through gene rearrangement events on its linear mega-plasmids.

**TABLE 1:** The quality metrics of sequencing and assembly for three *Rhodococcus* genomes

Strain name	DK17	DK176^*a*^	DK180^*a*^
Isolation source	Crude oil-contaminated soil	Crude oil-contaminated soil	Crude oil-contaminated soil
BioProject accession	PRJNA514400	PRJNA514400	PRJNA514400
BioSample accession	SAMN33169483	SAMN33169484	SAMN33169485
Assembly accession	GCA_038447505.2	GCA_038447315.2	GCA_038447005.2
No. of reads (Nanopore)	84,646	75,348	77,812
No. of reads (Illumina)	22,148,976	21,274,612	22,318,698
No. of contigs	6	5	6
No. of genes	8,906	8,210	8,892
No. of protein-coding genes	8,514	7,894	8,501
Total length (Nanopore, bp)	1,000,000,919	1,000,014,075	1,000,003,627
Total length (Illumina, bp)	3,344,495,376	3,212,466,412	3,370,123,398
Total length (assembly, bp)	9,527,724	8,826,323	9,520,489
Chromosome	7,909,450	7,902,594	7,902,427
pDK3	778,981	401,200	745,036
pDK1	398,449	398,884	398,391
pDK2	317,199	0	350,990
pDK4	109,875	109,875	109,875
pDK5	13,770	13,770	13,770
N50 (Nanopore, bp)	13,548	15,958	15,237
N50 (assembly, bp)	7,909,307	7,902,427	7,902,321
GC (%)	67.09	67.25	67.09
Genome coverage	243.56X	395.68X	337.58X
Completeness (%)	99.59	99.59	99.59
Contamination (%)	2.01	1.52	2.01

^*a*^ UV-induced mutant strain derived from DK17.

## Data Availability

All genomic sequences have been deposited in DDBJ/ENA/GenBank under the BioProject PRJNA514400 with the following accession numbers: DK17, GCA_038447505.2; DK176, GCA_038447315.2; and DK180, GCA_038447005.2 .
